# Famine in the Young and Risk of Later Hospitalization for COPD and Asthma

**DOI:** 10.1371/journal.pone.0082636

**Published:** 2013-12-23

**Authors:** Annet F. M. van Abeelen, Sjoerd G. Elias, Pim A. de Jong, Diederick E. Grobbee, Patrick M. M. Bossuyt, Yvonne T. van der Schouw, Tessa J. Roseboom, Cuno S. P. M. Uiterwaal

**Affiliations:** 1 Julius Center for Health Sciences and Primary Care, University Medical Center Utrecht, Utrecht, The Netherlands; 2 Department of Clinical Epidemiology, Biostatistics and Bioinformatics, Academic Medical Center, University of Amsterdam, Amsterdam, The Netherlands; 3 Department of Radiology, University Medical Center Utrecht, Utrecht, The Netherlands; 4 Department of Obstetrics and Gynecology, Academic Medical Center, University of Amsterdam, Amsterdam, The Netherlands; Baylor College of Medicine, United States of America

## Abstract

**Background:**

Undernutrition during critical periods of growth and development may permanently affect lung physiology and function.

**Objectives:**

To investigate whether acute undernutrition in childhood or young adulthood increases the risk of later hospitalization for obstructive airways disease, chronic obstructive pulmonary disease (COPD), or asthma.

**Methods:**

We studied 7,841 women from Prospect-EPIC who experienced the 1944–45 Dutch famine between ages 0 and 21. Pulmonary outcomes were measured by registered hospital admissions and exposure-blinded computed tomography (CT) in a subgroup of 295 women. With Cox proportional hazard regression we explored effects of famine exposure on risk of hospitalization for obstructive airways disease, COPD, and asthma. With logistic regression we explored effects of famine on risk of CT evidence of pulmonary disease.

**Results:**

Risks of hospitalization for obstructive airways disease, COPD, and asthma were increased after moderate famine exposure, and significantly increased after severe famine exposure: hazard ratios for obstructive airways disease were 1.31 (95% CI: 0.97 to 1.77) and 1.57 (95% CI: 1.10 to 2.23) respectively. Associations between famine exposure and hospitalization for COPD were stronger in ever-smokers than in never-smokers.

**Conclusions:**

Acute undernutrition in childhood or young adulthood is associated with an increased risk of later COPD and asthma hospitalization, possibly through increased sensitivity for tobacco smoke.

## Introduction

Respiratory disease is one of the leading causes of non-communicable disease mortality worldwide. In 2008, 4.2 million people died from respiratory diseases globally, accounting for 7% of the total and 12% of the non-communicable disease mortality [1].

There is increasing evidence suggesting that respiratory diseases originate in early life. Undernutrition during critical periods of development, including fetal life, infancy, and childhood, may result in permanent changes in the structure and physiology of the body [2].

Animal studies have demonstrated that starvation in rats resulted in decreased tissue elasticity in the lungs, which was not reversed after resumption of feeding [3], [4]. Furthermore, a study in rabbits has demonstrated that seven days of postnatal malnutrition results in reduced lung weight, lung/birth weight ratio, and number of alveoli and elastic fibers and collagen deposition [5].

A meta-analysis of eight studies showed that birth weight was positively associated with lung capacity [6]. In these studies however, birth weight served as a crude marker of fetal growth as a reflection of the fetal environment. The Dutch Famine Birth Cohort Study directly assessed the long-term effects of acute prenatal undernutrition on atopy, lung function, and obstructive airways disease in adult life [7]. The prevalence of obstructive airways disease was markedly increased in people exposed to famine in mid gestation, the period during which the bronchial tree grows most rapidly [7].

Recently, it has been postulated that alveolarization is not restricted to fetal life and early childhood, but that neoalveolarization continues through childhood and adolescence [8]. Therefore, undernutrition during these periods may affect the development of the lungs and consequently lead to morphological or physiological alterations. However, in contrast to the prenatal period, much less is known about later life pulmonary consequences from a disturbed growth and development in the postnatal period.

As far as we know, there are no individual subject exposure data showing a direct relation between undernutrition during postnatal development and the risk of obstructive airways disease, chronic obstructive pulmonary disease (COPD), or asthma. Here we report on the association between moderate and severe undernutrition during childhood or young adulthood and the risk of hospitalization for obstructive airways disease, and two of its component diseases COPD and asthma. In Text S1 we also report on associations between undernutrition and signs of pulmonary disease on computed tomography (CT)-scans in a subgroup. For this study, we used the Prospect-EPIC cohort data with individual information on exposure to the 1944–1945 Dutch famine. Since smoking is the most important single causal factor for developing COPD [9], we also investigated the association between undernutrition and the risk of hospitalization for COPD separately for never-smokers and ever-smokers.

## Subjects and Methods

### Ethics statement

All women signed informed consent before study inclusion. The study complies with the Declaration of Helsinki and was approved by the Institutional Review Board of the University Medical Center Utrecht.

### The Prospect-EPIC cohort

The Prospect-EPIC cohort consists of 17,357 women aged 49–70 years at recruitment between 1993 and 1997 (response rate 35%). It is part of the European Prospective Investigation into Cancer and nutrition (EPIC). The rationale and design of both EPIC and Prospect-EPIC have been described in detail elsewhere [10], [11]. Briefly, women residing in Utrecht or its vicinity were recruited through a breast cancer screening program.

At enrolment, participants were asked to return a general questionnaire about demographic and lifestyle factors and past and current morbidity, including smoking and level of education. Furthermore, trained assistants measured height, weight, and waist and hip circumferences, and checked the questionnaire for missing information. In addition, 573 women were randomly selected to undergo CT. Details are provided in Text S1.

### Famine exposure

#### The Dutch famine

The Dutch famine was an acute six month period of severe starvation in the urban western part of the Netherlands at the end of World War II, lasting from October 1944 to May 1945. This famine evolved from an accumulation of circumstances. Liberation of the Northern part came to a halt when the attack to capture the Rhine bridge at Arnhem (operation ‘Market Garden’) failed. In order to support the Allied offensive, the Dutch government in exile arranged a railroad strike to thwart German transport of troops and ammunition. As a reprisal, the German occupier banned all food transports. At the height of the famine, from December 1944 to April 1945, the official daily rations varied between 400 and 800 kcal [12]. The relative amount of proteins, fats, and carbohydrates remained essentially unchanged during this period [13]. After liberation on May 5th 1945, the food situation improved swiftly, ending the famine abruptly.

#### Famine exposure assessment

At the time of enrolment, participants filled out the general questionnaire, which contained questions about place of residence, and experiences of hunger and weight loss during the Dutch famine. Possible responses to these last two famine questions were: ‘hardly’, ‘little’, or ‘very much’. Responses ‘not applicable’ or ‘I don’t know’ to one or both famine questions were excluded. Responses were combined into a three-point subjective hunger score: ‘very much exposed’ to both hunger and weight loss was categorized as ‘severely exposed’, ‘hardly exposed’ to both hunger and weight loss as ‘unexposed’, the rest as ‘moderately exposed’.

#### Exposure age categories

Age at famine exposure was assessed taking October 1, 1944, the start of the famine, as reference. Exposure age was classified into three categories; childhood (0 to 9 years), adolescence (10 to 17 years), and young adulthood (18 years or older), according to the seven stages in the postnatal human life cycle as defined by Bogin [14]. We defined pre-adolescent childhood, a period of rapid growth with many developmental milestones in physiology, behaviour, and cognition, as the period between 0 and 9 years, just before the growth spurt in women [14], [15]. From the start of the growth spurt, at around 10 years, through age 17 is called adolescence [14], [15]. This period is characterized by the growth spurt including sexual development [14], [15]. From 18 years of age, we considered persons as young adults gradually reaching homeostasis in physiology.

### Subject selection

We excluded women born after the famine (*n* = 2,559) and those who resided outside occupied Netherlands during the famine (*n* = 1,732). Thus, both famine exposed and not exposed women in our study resided in occupied territory to ensure no differences in stress levels dependent of whether someone lived in an occupied or non-occupied territory. For 8,091 of the remaining 13,066 women the hunger score could be calculated (62%). Women not permitting data retrieval from the municipal administration registries, the National Medical registry, or Statistics Netherlands (*n* = 246) and women who had an unreliable date of hospital discharge diagnosis (*n* = 4) were also excluded, leaving 7,841 women for our analyses.

Out of the 573 women who underwent CT, we included 295 women for our analyses. A detailed description of subject selection for CT is provided in Text S1.

### Outcome assessment

Data on obstructive airways disease, COPD, and asthma events until 31 December 2007 were provided by linking the cohort with the National Medical Registry (hospital discharge diagnosis) and with Statistics Netherlands (cause of death). Events were coded according to the International Classification of Disease (ICD) coding system version 9 or 10 (main or subdiagnosis): (i) hospital admission ICD-9 codes 490–496 for obstructive airways disease, 491–492 and 496 for COPD, and 493 for asthma; (ii) cause of death ICD-10 codes J40–J47 and J67 for obstructive airways disease, J41–J44 for COPD, and J45–J46 for asthma.

### Data analysis

Participant characteristics at enrolment were first tabulated against severity of famine exposure to evaluate potential confounders.

We used Cox proportional hazard regression models to assess the effect of famine on the risk of hospitalization for obstructive airways disease, COPD, and asthma separately. To assess sensitive growth periods during female development in which undernutrition has the largest effect on later risk of hospitalization for obstructive airways disease, COPD, or asthma, we tested for interaction by introducing the cross-products of the famine score and age at start of the famine to the various models. Using logistic regression analyses, we assessed the association between famine exposure and the risk of CT evidence of pulmonary disease (presence of emphysema, airway wall thickening, or bronchiectasis; a more detailed description is provided in Text S1. Both models were used for adjustment for potential confounders, including age at start of the famine, smoking (never/past/current smoker and pack years), and level of education (low/intermediate/high; socioeconomic status proxy). Since undernutrition may affect susceptibility to environmental influences such as smoking, we analysed the association between famine exposure and hospitalization for COPD and CT evidence of pulmonary disease separately among never-smokers and ever-smokers.

We performed all statistical analyses with SPSS Statistics version 17.0 (SPPS, Chicago, IL, USA). *P*-values were based on two-sided tests with a cut-off level for statistical significance of 0.05. A detailed description of data analysis is provided in Text S1.

## Results

At the end of follow-up on 1 January 2008, 7,126 (91%) women were still alive, 666 (8%) had died, and 49 (1%) were lost to follow-up. During follow-up, a total of 247 (3%) had a severe episode of obstructive airways disease (238 hospital admissions and 33 deaths; 558,425 observation years), 213 women had a severe episode of COPD (204 hospital admissions and 33 deaths; 558,719 observation years), and 49 women had a severe episode of asthma (49 hospital admissions and 0 deaths; 559,651 observation years).

Table 1 shows baseline characteristics at recruitment. Overall, severely famine exposed women were on average older at the start of the famine, had higher BMI, waist circumference, and smoked more than unexposed women.

**Table 1:** Baseline characteristics of the study population according to level of famine exposure (none, moderate, or severe).

	Level of famine exposure		
	*None*	*Moderate*	*Severe*
**Number (%)**	3,576 (46)	2,974 (38)	1,291 (16)
**General characteristics**			
Age at start of the famine (years)^a^	8.3 (0 to 20.7)	9.5 (0 to 20.7)	10.1 (0 to 20.7)
Age at recruitment (years)^a^	59.0 (49.2 to 70.1)	60.4 (49.2 to 70.1)	60.8 (49.4 to 69.8)
**Body size**			
Height (cm)^b^	164.5 (5.9)	164.1 (6.0)	163.8 (6.2)
Weight (kg)^b^	70.3 (11.1)	70.8 (11.5)	70.7 (11.9)
Body mass index (kg/m^2^)^b^	26.0 (4.0)	26.3 (4.1)	26.4 (4.2)
Waist (cm)^b^	83.7 (9.8)	84.7 (10.0)	85.0 (10.4)
Hip (cm)^b^	105.9 (8.2)	106.1 (8.5)	105.9 (8.6)
Waist to hip ratio^b^	0.79 (0.06)	0.80 (0.06)	0.80 (0.06)
**Lifestyle**			
Level of education (%)			
- Low	1,798 (50)	1,358 (46)	650 (50)
- Intermediate	1,296 (36)	1,151 (39)	488 (38)
- High	477 (13)	463 (16)	153 (12)
Smoking (%)			
- Never	1,747 (49)	1,318 (44)	526 (41)
- Ever (current or past)	1,824 (51)	1,647 (56)	756 (59)
Smoking (pack years)^b^	5.6 (9.2)	6.7 (10.1)	8.0 (11.0)

a Median (min-max).

b Mean (standard deviation).

Table 2 shows that overall, the risk of hospitalization for obstructive airways disease and COPD was significantly higher among the moderately and severely famine exposed, in a dose-dependent manner. The risk of hospitalization for asthma was higher among moderately exposed and significantly higher among severely exposed women, also in a dose-dependent manner. Adjustment for age at start of the famine, smoking, and level of education as a proxy for socioeconomic status attenuated the risk estimates for obstructive airways disease and COPD. In the moderately exposed the risk of hospitalization for obstructive airways disease was 31% higher and in the severely exposed it was 57% higher than in unexposed women. For COPD these risk estimates were 32% and 53% for moderately and severely famine exposed women respectively (Table 2). Confounder adjustments did not change the risk estimates for asthma. The risk of hospitalization for asthma was 43% higher among moderately and 112% higher among severely famine exposed compared with unexposed women. Additional adjustment for BMI did not change these results (data not shown).

**Table 2:** (Un)adjusted hazard ratios and 95% confidence intervals (CI) for the risk of hospitalization for obstructive airways disease, COPD, and asthma in later life for all women (all ages; three exposure age categories combined) who reported to be moderately or severely famine exposed compared to those who reported to be unexposed to famine.

			Crude model		Adjusted model	
Level of famine exposure	Observation years	Number of cases	HR	95% CI	HR	95% CI
**Obstructive airways disease**						
Unexposed	253,057	81	1.00	reference	1.00	reference
Moderately exposed	212,898	102	1.40	1.05 to 1.88	1.31	0.97 to 1.77
Severely exposed	92,470	55	1.74	1.24 to 2.46	1.57	1.10 to 2.23
*P* for trend			0.001		0.009	
**COPD**						
Unexposed	253,165	68	1.00	reference	1.00	reference
Moderately exposed	213,008	89	1.45	1.06 to 1.99	1.32	0.95 to 1.82
Severely exposed	92,546	47	1.76	1.22 to 2.56	1.53	1.05 to 2.24
*P* for trend			0.002		0.02	
**Asthma**						
Unexposed	253,480	16	1.00	reference	1.00	reference
Moderately exposed	213,437	20	1.43	0.74 to 2.76	1.43	0.72 to 2.82
Severely exposed	92,733	13	2.11	1.02 to 4.39	2.12	1.00 to 4.49
*P* for trend			0.05		0.05	

Adjusted model: Adjusted for age at start of the famine (October 1, 1944), smoking (never/past/current and pack years), and level of education (low/intermediate/high; socioeconomic status proxy).

**Figure 1:**
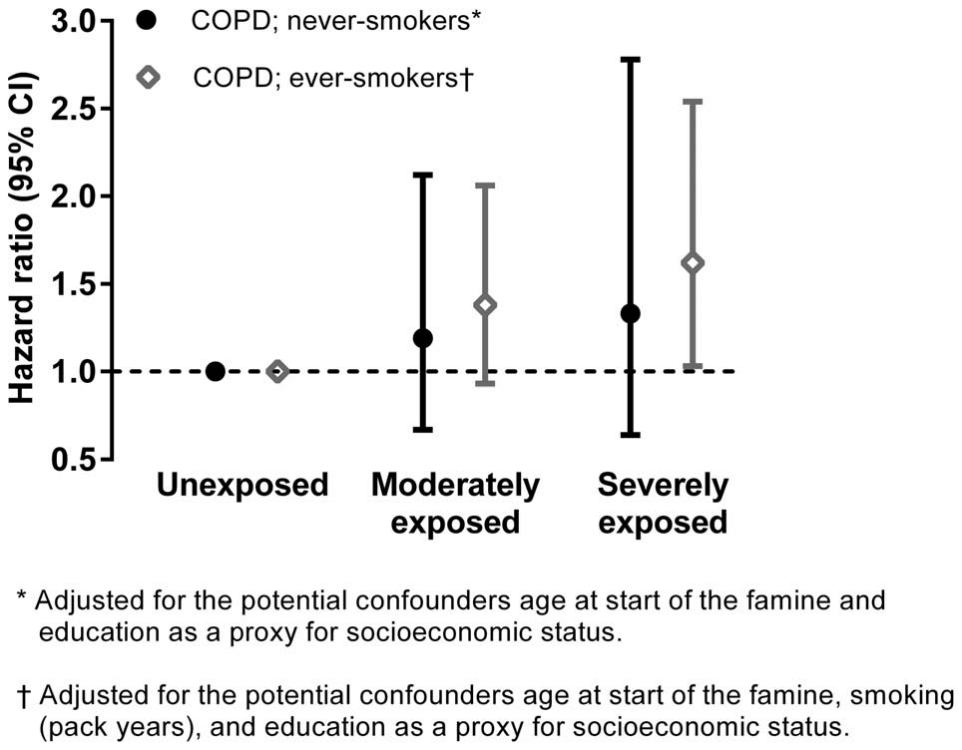
Famine and risk of later hospitalization for COPD among never-smokers and ever-smokers: Cox regression analysis. Note below Figure 1: Adjusted hazard ratios and 95% confidence intervals (CI) for the risk of hospitalization for COPD among never-smokers and ever-smokers for women who reported to be moderately or severely exposed to famine compared to those who reported to be unexposed to famine.

We did not find a statistically significant interaction between famine exposure and smoking nor between famine exposure and age at start of the famine. Figure 1 shows that among never-smokers, the risk of hospitalization for COPD (adjusted for age at start of the famine and education) among moderately famine exposed was 19% higher (95% CI: 0.67 to 2.12) and among severely famine exposed it was 33% higher (95% CI: 0.64 to 2.78) than in unexposed women (*P* for trend  = 0.40). Among ever-smokers these risk estimates were stronger. The risk of hospitalization for COPD (adjusted for age at start of the famine, smoking, and education) among moderately famine exposed was 38% higher (95% CI: 0.93 to 2.06) and among severely famine exposed it was 62% higher (95% CI: 1.03 to 2.54) than in unexposed women (*P* for trend  = 0.03).

Tables 3, 4, and 5 show the relation between famine exposure and subsequent obstructive airways disease, COPD, and asthma hospitalization risk within the exposure age categories. These tables show the crude results and adjusted for the potential confounders age at start of the famine, smoking, and level of education. Although there was no statistically significant interaction between famine exposure and age, we found a statistically significant dose-response relation between the severity of famine exposure and the risk of hospitalization for obstructive airways disease, COPD, and asthma within women aged 10–17 years at start of the famine. The dose-response relations for obstructive airways disease and asthma even remained statistically significant after adjustment for confounding.

**Table 3:** (Un)adjusted hazard ratios and 95% confidence intervals (CI) for the risk of hospitalization for obstructive airways disease for women within each of the exposure age categories: 0–9 years, 10–17 years, and ≥18 years who reported to be moderately or severely famine exposed compared to those who reported to be unexposed to famine.

		Crude model		Multivariable model 1	
Age at famine categories	Number of cases	Hazard ratio	95% CI	Hazard ratio	95% CI
**0 to 9 years**					
Unexposed	37	1.00	reference	1.00	reference
Moderately exposed	39	1.36	0.87 to 2.14	1.34	0.84 to 2.14
Severely exposed	16	1.31	0.73 to 2.36	1.29	0.71 to 2.35
*P* for trend		0.23		0.28	
**10 to 17 years**					
Unexposed	38	1.00	reference	1.00	reference
Moderately exposed	48	1.37	0.89 to 2.09	1.22	0.79 to 1.89
Severely exposed	33	2.10	1.32 to 3.35	1.80	1.11 to 2.90
*P* for trend		0.002		0.02	
**≥18 years**					
Unexposed	6	1.00	reference	1.00	reference
Moderately exposed	15	2.14	0.83 to 5.51	1.53	0.56 to 4.18
Severely exposed	6	2.18	0.70 to 6.76	1.52	0.45 to 5.07
*P* for trend		0.14		0.48	

Multivariable model 1: Adjusted for age at start of the famine (October 1, 1944), smoking (never/past/current smoker and pack years), and level of education (low/intermediate/high; socioeconomic status proxy).

**Table 4:** (Un)adjusted hazard ratios and 95% confidence intervals (CI) for the risk of hospitalization for COPD for women within each of the exposure age categories: 0–9 years, 10–17 years, and ≥18 years who reported to be moderately or severely exposed to famine compared to those who reported to be unexposed to famine.

		Crude model		Multivariable model 1	
Age at famine categories	Number of cases	Hazard ratio	95% CI	Hazard ratio	95% CI
**0 to 9 years**					
Unexposed	30	1.00	reference	1.00	reference
Moderately exposed	34	1.47	0.90 to 2.40	1.39	0.83 to 2.30
Severely exposed	14	1.42	0.75 to 2.68	1.35	0.71 to 2.57
*P* for trend		0.17		0.25	
**10 to 17 years**					
Unexposed	34	1.00	reference	1.00	reference
Moderately exposed	40	1.27	0.80 to 2.00	1.12	0.70 to 1.79
Severely exposed	28	1.99	1.21 to 3.28	1.66	0.99 to 2.77
*P* for trend		0.009		0.07	
**≥18 years**					
Unexposed	4	1.00	reference	1.00	reference
Moderately exposed	15	3.24	1.07 to 9.76	2.31	0.73 to 7.35
Severely exposed	5	2.71	0.73 to 10.09	1.81	0.45 to 7.28
*P* for trend		0.10		0.40	

Multivariable model 1: Adjusted for age at start of the famine (October 1, 1944), smoking (never/past/current smoker and pack years), and level of education (low/intermediate/high; socioeconomic status proxy).

**Table 5:** (Un)adjusted hazard ratios and 95% confidence intervals (CI) for the risk of hospitalization for asthma for women within each of the exposure age categories: 0–9 years, 10–17 years, and ≥18 years who reported to be moderately or severely exposed to famine compared to those who reported to be unexposed to famine.

		Crude model		Multivariable model 1	
Age at famine categories	Number of cases	Hazard ratio	95% CI	Hazard ratio	95% CI
**0 to 9 years**					
Unexposed	9	1.00	reference	1.00	reference
Moderately exposed	7	1.01	0.38 to 2.71	1.00	0.35 to 2.90
Severely exposed	4	1.37	0.42 to 4.46	1.55	0.46 to 5.21
*P* for trend		0.66		0.55	
**10 to 17 years**					
Unexposed	6	1.00	reference	1.00	reference
Moderately exposed	12	2.19	0.82 to 5.84	2.06	0.77 to 5.53
Severely exposed	8	3.14	1.09 to 9.06	2.86	0.98 to 8.29
*P* for trend		0.03		0.05	
**≥18 years**					
Unexposed	1	1.00	reference	1.00	reference
Moderately exposed	1	0.84	0.05 to 13.48	0.80	0.05 to 13.81
Severely exposed	1	2.19	0.14 to 35.05	1.68	0.08 to 33.70
*P* for trend		0.62		0.75	

Multivariable model 1: Adjusted for age at start of the famine (October 1, 1944), smoking (never/past/current smoker and pack years), and level of education (low/intermediate/high; socioeconomic status proxy).

The risk of CT evidence of pulmonary disease seemed higher among moderately and severely exposed women, although not statistically significant. Especially among ever-smokers, we found a statistically significant dose-response relation between famine exposure and the risk of CT evidence of pulmonary disease. More detailed results with regard to CT evidence of pulmonary disease are shown in Table S1.

## Discussion

This study demonstrates for the first time that a relatively short period of moderate or severe undernutrition during childhood or young adulthood is associated with an increased risk of hospitalization for obstructive airways disease, COPD, and asthma in adult life, in a dose-dependent manner. The effect of famine on the risk of hospitalization for COPD seems to be stronger among ever-smokers than among never-smokers. These findings were confirmed by CTs of the lungs of a random subset of participants of the Prospect-EPIC study. In these analyses, the association between famine exposure and CT evidence of pulmonary disease was also stronger among ever-smokers, suggesting an early life – later environment interaction.

Before further discussion, some aspects of our study require consideration. The Dutch famine is a ‘natural experiment’ in history, which gave us the unique possibility to study the long-term effects of acute undernutrition during childhood, adolescence, and young adulthood in otherwise well-nourished girls and women. We used individual famine exposure data in order to enhance the precision of exposure measurement. The drawback of individual data may be its subjective nature. However, our exposure classification agrees with rationing practices at that time. The allocated individual amount of calories was based on age. Young children (1–3 years) were relatively protected from the famine and received about 50%, whereas adults received about 25% of the distributed amount of calories at the start of the famine [12]. These historical facts are reflected by our data, showing that the older women were at the start of the famine, the higher the proportion that reported to have been exposed to famine. Furthermore, the famine was worst in large cities in the Western part of the Netherlands, which is also reflected in our data. The percentage of women who reported to be severely exposed to famine was 12% in those residing in the Western part of the Netherlands, whereas it was 4% for those residing in the Eastern part of the Netherlands. This may be considered in support of the quality of our exposure data. Nevertheless, our individual famine score is still susceptible for misclassification, since it was based on recollection. Especially this may be true for the youngest age group. Although, it is conceivable these young women have learned about their famine experiences from their parents and family. Nevertheless, if recall in the youngest age group during the famine is not as good as in the older women, this would lead to larger exposure misclassification in that age group. However, this misclassification is unlikely to be related to the outcome in this study and would therefore lead to an underestimation of the true association.

Selection bias might be another issue. The findings in this study are conditional on survival until examination between 1993 and 1997. This could have affected our results, but we can only speculate about this. An estimated 22,000 deaths are caused directly by the famine on an estimated population of 4.3 million people who suffered from the famine [16], [17]. Of these fatalities, an estimated 75% were male and 79% were babies or over 65 years of age [16]. Since our study only consists of females and less than 10% are babies, we suspect a minimal direct increase in mortality due to the famine in the source population of our study. Also, mortality before study inclusion at the age of 50 due to obstructive airways disease among women is also very low. To illustrate this; only 34 out of 5,419,598 women aged between 0 and 50 years died due to obstructive airways disease in 1996 [18], [19]. Thus, famine survivors may constitute of a group of women with somewhat better social resources for health and constitution, since the famine may have led to a somewhat increased mortality especially in women and girls with less efficient metabolism and low fat-reserves. However, the above makes the extent of potential selective survival before study inclusion negligible.

We believe we have captured real undernutrition as the determinant of later life outcome with these individual self-reports of famine exposure. However, we cannot exclude that these measures are proxies of other phenomena such as psychological stress, as with all retrospective studies on wartime famine exposure.

Since we identified our cases by means of linkage with the National Medical Registry (hospital discharge diagnosis), we probably included the more severe COPD and asthma cases. As a result, we cannot conclude from this study whether undernutrition during postnatal development leads to an increased incidence of respiratory diseases, to a more severe course of the diseases or to a combination of both.

We studied only women, recruited through a breast cancer screening program. Since there is an increasing body of evidence showing sex specific differences in programming, the generalizability of these results to men is unknown [20].

We found a significant dose-dependent association between famine exposure and the risk of hospitalization for obstructive airways disease, COPD, and asthma in adult life. These findings were confirmed by the CTs. We demonstrated that with increasing famine exposure the risk of CT evidence of pulmonary disease also seem to increase, although we could not demonstrate a statistically significant dose-response relation. This may be due to the small number of cases, since CTs were available for only 295 women.

Analysing the effects of famine on the risk of hospitalization for COPD separately among never-smokers and ever-smokers resulted in stronger associations among ever-smokers. This finding was also confirmed by the CTs. We found a statistically significant dose-response relation between famine exposure and the risk of CT evidence of pulmonary disease among ever-smokers, while among never-smokers we could not demonstrate a dose-response relation. These results might suggest that women who were famine exposed during their childhood, adolescence, or young adulthood were more sensitive for the toxic effects of smoking. As a consequence, the combination of early famine exposure and smoking later on results in the highest risk of hospitalization for COPD in adult life. We consider it very unlikely that these results are due to residual confounding. We measured lifestyle factors, including smoking, with as much precision as possible. We accurately adjusted the analyses within the ever-smokers group for pack years. Furthermore, when adjusting for covariables in the various regression models, we took special care to provide accurate fit of the data. However, we cannot completely exclude the possibility of residual confounding.

We could not demonstrate a statistically significant interaction between the effects of age at start of the famine and famine exposure. However, we found a statistically significant dose-response relation between the effects of famine exposure on the risk of hospitalization for obstructive airways disease, COPD, and asthma within the 10–17 year age category, while no significant dose-response relations were found in the 0–9 year and ≥18 year exposure age categories. One possible explanation for the fact that we could not demonstrate an association between famine exposure and the risk of hospitalization for obstructive airways disease, COPD, or asthma in young childhood could be that early childhood exposure may have been less severe than in older age groups. Young children were relatively protected from the famine within families and by special committees such as the Interchurch Organization [12], [16]. This could have resulted in lower exposure contrast and less power to detect an association in the youngest age group. However, this is in contrast with the previous finding of a higher risk of breast cancer after severe famine exposure in this age group [21]. Since we could not demonstrate a statistically significant interaction, further research into the age specific effects is needed to confirm these findings.

As far as we know there is no previous evidence on the long-term effects of postnatal undernutrition on adult lung function. The results of this study, however, support the autophagy hypothesis. Hereby, critical events like starvation induce adaptation processes by increased activation of autophagy, which in turn is associated with an increased risk of COPD [22]. Besides, a study in anorexia patients has demonstrated an association between chronic malnourishment and emphysema-like changes in the lungs of these patients [23]. The results of the present study agree with and expand the existing literature that besides chronic malnutrition, short-term undernutrition during childhood growth and development also results in an increased risk of hospitalization for obstructive airways disease, COPD, and asthma.

### Relevance

Our findings indicate that moderate or severe undernutrition during childhood or young adulthood is associated with an increased risk of hospitalization for obstructive airways disease, COPD, and asthma in adult life. Famine and undernutrition are still a major problem worldwide; never before have there been so many hungry people in the world [24]. Our findings suggest that famine may not only have short term but also long term consequences especially for those who experienced famine in early life, making them more susceptible to adverse environmental influences, such as smoking.

### Conclusions

This study provides the first direct evidence that a short period of moderate or severe undernutrition during postnatal development increases the risk of hospitalization for obstructive airways disease, COPD, and asthma in adult life, which may be due to an increased vulnerability for the toxic effects of smoking.

## Supporting Information

Text S1 **Online supplementary text file – methods.**
(DOC)

Table S1 **Online supplementary table file – results, tables 6 and 7.**
(DOC)
