# Supplementary material for: Famine in the Young and Risk of Later Hospitalization for COPD and Asthma
Source: PLoS One. 2013 Dec 23;8(12):e82636. doi: 10.1371/journal.pone.0082636 (PMC3871614; doi:10.1371/journal.pone.0082636)
Supplement: Text S1 — Online supplementary text file – methods. (DOC) [file pone.0082636.s001.doc]

**Subject selection for CT**

Of the 573 women who underwent CT, we excluded women born after the famine (*n*=53), women who resided outside occupied Netherlands during the famine (*n*=48), women for whom no hunger score could be calculated (*n*=171), and women not permitting data retrieval from the municipal administration registries, the National Medical Registry, or Statistics Netherlands (*n*=6), leaving 295 women for our analyses.

**Outcome assessment for CT**

We used a prospective ECG-gated unenhanced CT. A 16-slice scanner (Mx 8000 IDT 16; Philips Medical Systems, Best, the Netherlands) with 0.42-second rotation time was used to obtain 1.5-mm thick sections in one breath hold from the level of the tracheal bifurcation to below the base of the heart. At these levels, the lungs were also visible and these parts of the lungs were assessed for the presence of emphysema, airway wall thickening, and bronchiectasis by an experienced chest radiologist (PAdJ) [1,2], blinded for the level of famine exposure. If one or more of these abnormalities were present, this participant was classified as having any pulmonary disease manifestation.

**Data analysis**

Prospect-EPIC participant characteristics at enrolment, including demographics, anthropometry, and lifestyle, were first tabulated against severity of famine exposure in order to evaluate potential confounders. We used Cox proportional hazard regression models to assess the effect of famine exposure on the risk of hospitalization for obstructive airways disease, COPD, and asthma separately. We evaluated the effect of famine on the risk of hospitalization and we also defined composite outcomes as disease occurrence either manifested by hospitalization or death. Follow-up time was defined as the time from date of birth to the event of interest. Follow-up times of subjects who remained event-of-interest free were considered censored at the date of death due to other causes, the date of lost to follow-up, or on 1 January 2008, whichever came first. Women with manifest COPD and asthma contributed to both the COPD and the asthma analyses, but with follow-up times matching the respective outcomes. In the obstructive airways disease analyses, these women contributed with follow-up times matching the first date of hospital admission due to either COPD or asthma. We used trend tests to explore dose-response relations by introducing the famine exposure score as an ordinal variable (one for ‘unexposed’, two for ‘moderately exposed’, and three for ‘severely exposed’). To assess sensitive growth periods, we tested for interaction by introducing the cross-products of the famine score and age at start of the famine to the various models.

First, we analyzed the crude association between famine exposure and hospitalization for obstructive airways disease, COPD, and asthma. In a second model, we adjusted for the potential confounders age at start of the famine (years), smoking (never/past/current smoker and pack years), and level of education (low/intermediate/high; socioeconomic status proxy). Since smoking is the most important risk factor for COPD, we tested for interaction by introducing the cross-products of the famine score and smoking to the various models. We analyzed the association between famine exposure and the risk of hospitalization for COPD separately among never-smokers and ever-smokers (current smokers and those who had ever smoked before the time of assessment).

Using logistic regression analyses, we assessed the association between famine exposure and the risk of CT evidence of pulmonary disease. We adjusted for potential confounders, including age at start of the famine, smoking (never/past/current smoker and pack years), and level of education (low/intermediate/high; socioeconomic status proxy). To assess sensitive growth periods, we tested for interaction by introducing the cross-products of the famine score and age at start of the famine to the various models. To assess effect modification by smoking, we tested for interaction by introducing the cross-products of the famine score and smoking to the various models. We analyzed the association between famine exposure and CT evidence of pulmonary disease separately among never-smokers and ever-smokers.

Continuous variables were introduced as such in the different models; for categorical variables, we created indicator variables. To include pack years and smoking (never/past/current) simultaneously in the models, we used the pack years centered around its mean multiplied by smoking (3). For the Cox proportional hazard regression analyses, we evaluated the proportionality of the hazards over time with log minus log plots. Results are reported as hazard ratios (HR) with 95% confidence intervals (CI) between those who were unexposed to famine compared to those who were moderately or severely famine exposed. For the logistic regression analyses, results are reported as odds ratios (OR) with 95% CI between those who were unexposed to famine compared to those who were moderately or severely famine exposed. We performed all statistical analyses with SPSS Statistics version 17.0 (SPPS, Chicago, IL, USA). P-values were based on two-sided tests with a cut-off level for statistical significance of 0.05.

**References**

1. De Jong PA, Muller NL, Pare PD, Coxson HO (2005) Computed tomographic imaging of the airways: relationship to structure and function. Eur Respir J 26: 140-152.
2. De Jong PA, Long FR, Nakano Y (2006) Computed tomography dose and variability of airway dimension measurements: how long can we go? Pediatr Radiol 36: 1043-1047.

3. Breslow N, Day N (1980) Statistical methods in cancer research. Volume I - The analysis of case-control studies. IARC Scientific Publications.
