# Supplementary material for: Famine in the Young and Risk of Later Hospitalization for COPD and Asthma
Source: PLoS One. 2013 Dec 23;8(12):e82636. doi: 10.1371/journal.pone.0082636 (PMC3871614; doi:10.1371/journal.pone.0082636)
Supplement: Table S1 — Online supplementary table file – results, tables 6 and 7. (DOC) [file pone.0082636.s002.doc]

**Results: CT evidence of pulmonary disease**

Table 6 shows that overall, the risk of CT evidence of pulmonary disease seemed higher among moderately and severely famine exposed women, although not statistically significant. Table 6 also shows that among never-smokers, there was no association (*P* for trend=0.62). Among ever-smokers, we found a statistically significant dose-response relation between famine exposure and the risk of CT evidence of pulmonary disease (*P* for trend=0.01). The risk of CT evidence of pulmonary disease was higher among moderately famine exposed (OR: 1.70) and significantly higher among severely famine exposed women (OR: 3.23). The *P* for interaction between famine exposure and smoking (ever/never) was 0.10. The *P* for interaction between severe famine exposure and ever-smoking was 0.04. There was no interaction between famine exposure and age at start of the famine. Age specific results for the association between famine exposure and the risk of CT-evidence of pulmonary disease are shown in Table 7.

**Online supplementary Tables 6 and 7**

**Table 6**. Odds ratios (OR) and 95% confidence intervals (CI) for the risk of CT evidence of pulmonary disease for women who reported to be moderately or severely exposed to famine compared to those who reported to be unexposed to famine.

|  |  |  | **Crude model** | | **Adjusted model** | |
| --- | --- | --- | --- | --- | --- | --- |
| Level of famine exposure | N Total | N Pulmonary disease | Odds ratio | 95% CI | Odds ratio | 95% CI |
| **Overall** |  |  |  |  |  |  |
| Unexposed | 143 | 60 | 1.00 | reference | 1.00 | reference |
| Moderately exposed | 106 | 52 | 1.33 | 0.80 to 2.21 | 1.23 | 0.72 to 2.08 |
| Severely exposed | 46 | 24 | 1.51 | 0.77 to 2.94 | 1.37 | 0.69 to 2.72 |
| *P* for trend |  |  | 0.16 | | 0.31 | |
| **According to cigarette smoking** |  |  |  |  |  |  |
| **Never-smokers** |  |  |  |  |  |  |
| Unexposed | 77 | 34 | 1.00 | reference | 1.00 | reference |
| Moderately exposed | 48 | 21 | 0.98 | 0.48 to 2.03 | 0.93 | 0.44 to 1.96 |
| Severely exposed | 24 | 9 | 0.76 | 0.30 to 1.94 | 0.69 | 0.26 to 1.80 |
| *P* for trend |  |  | 0.62 | | 0.48 | |
| **Ever-smokers** |  |  |  |  |  |  |
| Unexposed | 66 | 26 | 1.00 | reference | 1.00 | reference |
| Moderately exposed | 58 | 31 | 1.77 | 0.86 to 3.61 | 1.70 | 0.80 to 3.63 |
| Severely exposed | 22 | 15 | 3.30 | 1.18 to 9.18 | 3.23 | 1.11 to 9.43 |
| *P* for trend |  |  | 0.01 | | 0.02 | |

Adjusted model: Adjusted for age at start of the famine (October 1, 1944), smoking (never/past/current and pack years), and level of education (low/intermediate/high; socioeconomic status proxy

**Table 7. Odds ratios (OR) and 95% confidence intervals (CI) for the risk of CT evidence of pulmonary disease for women within each of the exposure age categories: 0-9 years, 10-17 years, and ≥18 years who reported to be moderately or severely exposed to famine compared to those who reported to be unexposed to famine.**

|  |  |  | **Crude model** | | **Adjusted model** | |
| --- | --- | --- | --- | --- | --- | --- |
| Level of famine exposure | N Total | N Pulmonary disease | Odds ratio | 95% CI | Odds ratio | 95% CI |
| **0 to 9 years** |  |  |  |  |  |  |
| Unexposed | 98 | 34 | 1.00 | reference | 1.00 | reference |
| Moderately exposed | 64 | 30 | 1.66 | 0.87 to 3.16 | 1.63 | 0.84 to 3.17 |
| Severely exposed | 29 | 14 | 1.76 | 0.76 to 4.06 | 1.66 | 0.69 to 4.00 |
| *P* for trend |  |  | 0.10 | | 0.14 | |
| **10 to 17 years** |  |  |  |  |  |  |
| Unexposed | 41 | 23 | 1.00 | reference | 1.00 | reference |
| Moderately exposed | 39 | 21 | 0.91 | 0.38 to 2.20 | 0.95 | 0.36 to 2.54 |
| Severely exposed | 15 | 8 | 0.89 | 0.27 to 2.93 | 0.72 | 0.20 to 2.59 |
| *P* for trend |  |  | 0.83 | | 0.65 | |
| **≥18 years** |  |  |  |  |  |  |
| Unexposed | 4 | 3 | 1.00 | reference | 1.00 | reference |
| Moderately exposed | 3 | 1 | 0.17 | 0.01 to 4.51 | * | * |
| Severely exposed | 2 | 2 | * | * | * | * |
| *P* for trend |  |  | 0.77 | | * | |

Adjusted model: Adjusted for age at start of the famine (October 1, 1944), smoking (never/past/current and pack years), and level of education (low/intermediate/high; socioeconomic status proxy).

* Since the number of cases were too small in this subgroup, ORs could not be calculated.
